# One Health Insights into Pulmonary Hypertension: Bridging Human and Canine Medicine

**DOI:** 10.3390/vetsci13040341

**Published:** 2026-03-31

**Authors:** Ana Reis-Ferreira, Joana Castanheira-Moreira, Helena Coelho-Pinho, Marta Mendes, Luís Lobo, Carmen Brás-Silva, Mário Santos, Ana Patrícia Fontes-Sousa

**Affiliations:** 1 School of Medicine and Biomedical Sciences, University of Porto, Rua Jorge de Viterbo Ferreira 228, 4050-313 Porto, Portugal; anasf.reis@gmail.com (A.R.-F.); joanacastanheiram@gmail.com (J.C.-M.); hpinho1212@gmail.com (H.C.-P.); marta_gmendes@hotmail.com (M.M.); 2 Hospital Veterinário do Porto, Travessa Silva Porto 174, 4250-475 Porto, Portugal; luis.lobo@onevetgroup.pt; 3 Faculty of Veterinary Medicine, Lusófona University, 1749-024 Lisboa, Portugal; 4 Center for Animal Science Studies (CECA), Instituto de Ciências, Tecnologias e Agroambiente (ICETA), University of Porto, 4051-401 Porto, Portugal; 5 RISE-Health, Faculdade de Medicina, Universidade do Porto, Alameda Prof. Hernâni Monteiro, 4200-319 Porto, Portugal; carmensb@med.up.pt; 6 Pulmonary Vascular Disease Unit, Department of Cardiology, Unidade Local de Saúde de Santo António, 4099-001 Porto, Portugal; massantos@icbas.up.pt; 7 Department of Immuno-Physiology and Pharmacology, Unidade Multidisciplinar de Investigação Biomédica (UMIB), Instituto de Ciências Biomédicas Abel Salazar (ICBAS), Universidade do Porto, 4050-313 Porto, Portugal; 8 ITR-Laboratory for Integrative and Translational Research in Population Health, Rua das Taipas 135, 4050-600 Porto, Portugal; 9 RISE-Health, Department of Immuno-Physiology and Pharmacology, Veterinary Hospital of the University of Porto (UPVET), School of Medicine and Biomedical Sciences, University of Porto, Rua Jorge de Viterbo Ferreira 228, 4050-313 Porto, Portugal

**Keywords:** comparative cardiology, echocardiography, one health, pulmonary hypertension, right heart failure, veterinary cardiology

## Abstract

Pulmonary hypertension is a progressive syndrome characterised by pulmonary vascular dysfunction, inflammation, maladaptive remodelling, and progressive right-ventricular strain. Translational progress remains limited because experimental models reproduce only selected aspects of the complexity of human disease. This narrative review evaluates naturally occurring canine disease as a comparative and spontaneous model of human pulmonary hypertension within a One Health framework. To achieve this, we synthesise recent human and veterinary literature, international consensus statements, and key registry and imaging studies. We outline current human definitions and diagnostic pathways based on right-heart catheterisation, together with the veterinary probability-based approach centred on echocardiography; compare epidemiology across species; and summarise contemporary mechanisms spanning vascular dysfunction, immune and metabolic signalling, and right-ventricular adaptation. We then examine canine conditions that parallel major human pulmonary hypertension phenotypes, including left-heart disease due to myxomatous mitral valve degeneration, fibrotic interstitial lung disease in West Highland White Terriers, sleep-related airway obstruction in brachycephalic breeds, and rare venous and capillary disorders. When combined, these spontaneous models provide opportunities to investigate disease-modifying techniques other than vasodilation and allow for the longitudinal, real-world evaluation of imaging, functional assessments, and circulating biomarkers. To improve care for both veterinary and human patients, we conclude by outlining priorities for mechanism-based clinical trials, shared outcome measures, prospective registries and biobanks, and harmonised definitions.

## Simple Summary

Pulmonary hypertension is a disorder in which pressure within the pulmonary circulation is persistently elevated, increasing the workload on the right side of the heart and predisposing affected individuals to heart failure. Despite advances, current drug therapy mainly targets vascular tone and does not reliably halt or reverse structural remodelling of the pulmonary vessels or the failing right ventricle. This review integrates current human and veterinary evidence through a One Health framework to highlight how naturally occurring disease in dogs can accelerate scientific discovery. Definitions, clinical characteristics, and diagnostic pathways are reviewed, with emphasis on canine models that closely recapitulate human disease processes, including pulmonary hypertension secondary to left-sided heart disease, fibrotic lung disease in West Highland White Terriers, sleep-related airway obstruction in brachycephalic breeds, and rare disorders affecting small pulmonary veins and capillaries. We outline practical opportunities for cross-species biomarkers, imaging endpoints, and therapeutic testing within real-world comorbidities. Overall, coordinated human–animal research has the potential to enhance early detection, refine risk stratification, and drive the development of truly disease-modifying therapies. Future progress will depend on harmonised cross-species definitions, prospective registries and biobanks, shared biomarkers and imaging endpoints, and collaborative translational studies built around naturally occurring disease.

## 1. Introduction

Pulmonary hypertension (PH) is a haemodynamic and pathophysiological condition of abnormally elevated pressure within the pulmonary circulation. In humans, PH is defined by a mean pulmonary arterial pressure (mPAP) >20 mmHg at rest measured by right-heart catheterisation (RHC). Pre-capillary PH is further defined by a pulmonary arterial wedge pressure (PAWP) ≤15 mmHg and pulmonary vascular resistance (PVR) >2 Wood units (WU), whereas post-capillary PH is identified by elevated left-sided filling pressures and may be further stratified according to PVR [1,2,3]. In dogs, invasive haemodynamic confirmation is rarely performed clinically; accordingly, the American College of Veterinary Internal Medicine (ACVIM) recommends a probability-based echocardiographic approach centred on peak tricuspid regurgitation velocity (TRV) interpreted together with supportive right-heart and pulmonary artery (PA) findings; this approach identifies dogs with an intermediate or high probability of PH (e.g., TRV > 3.4 m/s, or TRV 3.0–3.4 m/s with additional echocardiographic findings) [4]. As in human medicine, canine PH is a syndrome that can be clinically classified as either pre-capillary or post-capillary, distinctions that carry important diagnostic, prognostic, and therapeutic implications [4,5,6]. This classification is particularly relevant in myxomatous mitral valve disease (MMVD), the most common cause of post-capillary PH in dogs, where recent studies suggest a progression from a predominantly venous/post-capillary phenotype towards a mixed post- and pre-capillary profile associated with increased estimated PVR and secondary pulmonary vascular remodelling [6,7,8].

Across species, prognosis depends strongly on aetiology, haemodynamic severity, and right-ventricular (RV) adaptation. In a large European registry encompassing multiple human PH aetiologies, transplant-free five-year survival was approximately 54%, with poorer outcomes in patients with PH associated with left-heart disease (LHD) or advanced lung disease; although survival has improved in certain phenotypes of PH, such as pulmonary arterial hypertension (PAH), long-term mortality remains substantial [9]. In dogs, PH is likewise associated with adverse outcomes. In canine MMVD, both the presence and severity of PH are linked to reduced survival, and in cohorts with mixed aetiologies, markers of RV dysfunction, including reduced tricuspid annular plane systolic excursion, right atrial enlargement, and right-sided heart failure, are among the strongest predictors of mortality [10,11,12].

In humans, therapeutic progress has been greatest in PAH, where management has evolved from simple vasodilation to a risk-stratified, pathway-directed approach that now includes modulation of activin signalling [2,13]. By contrast, veterinary management remains less advanced and continues to rely largely on phosphodiesterase-5 inhibition alongside treatment of the underlying condition [4]. This disparity reflects a broader translational challenge: conventional experimental models reproduce only selected components of PH biology and fail to fully capture the chronicity, multimorbidity, and right-heart adaptation that characterise naturally occurring disease. For example, the monocrotaline rat model is confounded by extra-pulmonary toxicities and inconsistent plexiform pathology, whereas “two-hit” paradigms such as SU5416 combined with hypoxia (SuHx) better reproduce severe, persistent, angio-obliterative phenotypes, with emerging links to innate-immune signalling pathways (e.g., stimulator of interferon genes-NLRP3) [14,15,16]. In parallel, post-capillary/PH-LHD platforms are being refined; for example, left pulmonary vein banding induces pulmonary venous congestion with vascular remodelling and mild PH. Chronic hypoxia remains a useful model of vasoconstriction and muscularisation but typically regresses after re-oxygenation [14,17,18]. These limitations contribute to translational attrition and underscore the need for multi-platform strategies that integrate animal models with patient-derived systems to improve fidelity [14,19].

A One Health and comparative medicine perspective can help to narrow this translational gap. In dogs, standardised, probability-based echocardiographic criteria and clinically aligned classification systems enable shared biomarker evaluation, harmonised diagnostic approaches, and pragmatic therapeutic testing within real-world comorbidity settings. Together, these features position the dog as a natural and ethically spontaneous model that complements reductionist laboratory systems [4]. Comparative cardiology already provides strong precedents; for example, Doberman Pinschers with naturally occurring dilated cardiomyopathy closely recapitulate key human phenotypes and genetic risk, illustrating the potential for bi-directional discovery [20,21,22]. In this context, we propose that comparative medicine should be actively leveraged to advance PH biology and that naturally occurring canine disease should be prioritised as a preclinical platform capable of more faithfully reflecting human disease than traditional experimental models [4,23,24].

## 2. Classification of Pulmonary Hypertension

### 2.1. Clinical Classification

Clinical classification of PH provides a structured approach to organising the diverse conditions with elevated pulmonary arterial pressure (PAP) into groups that exhibit broadly similar pathobiological mechanisms, haemodynamic profiles, prognostic features, and therapeutic strategies. In humans, the five-group scheme is well established and was reaffirmed and updated by the 7th World Symposium (2024) [1]. In dogs, the ACVIM consensus proposes a cause-based framework that mirrors human principles but reflects species-specific aetiologies and clinical realities [4]. Table 1 presents a concise, consensus-aligned comparison of the human and canine classifications [1,2,4].

### 2.2. Pre-Capillary and Post-Capillary PH

PH can be subdivided into pre- and post-capillary forms according to RHC. Pre-capillary PH is characterised by elevated PAP with normal left-sided filling pressure and increased PVR, indicating disease centred in the pulmonary circulation. Post-capillary PH combines elevated PAP with elevated left-sided filling pressure, reflecting LHD; contemporary frameworks further separate isolated post-capillary PH from combined post-/pre-capillary PH phenotypes [1,2,25]. Histopathologically, isolated post-capillary PH primarily reflects passive backward transmission of elevated left-sided filling pressures, with pulmonary venous and capillary congestion [25,26]. With chronicity, however, the vascular phenotype may extend beyond the venous bed, and combined post-/pre-capillary PH is associated with a mixed vasculopathy that includes venous intimal fibrosis and “arterialisation”, capillary congestion, and secondary pulmonary arterial remodelling, including medial hypertrophy, intimal fibrosis/proliferation, and adventitial remodelling [25,26,27,28]. Human morphometric studies support this concept, showing that PH due to LHD is not purely venous but involves global pulmonary vascular remodelling, with severity correlating particularly with venous and small-vessel intimal thickening [27]. In contrast, PAH is a primary pre-capillary arteriopathy characterised by remodelling of the intima, media and adventitia, with plexiform lesions in advanced forms [28].

In dogs, the same physiological distinction is clinically important but is usually inferred non-invasively, as catheterisation is seldom performed [4]. Most canine post-capillary PH occurs secondary to MMVD or other forms of LHD and is therefore classified as group 2 PH [4,6]. Histopathological studies in dogs with MMVD-associated PH demonstrate pulmonary arterial medial remodelling driven by smooth-muscle cell hyperplasia/hypertrophy, together with collagen deposition, muscularisation of normally non-muscular small pulmonary arteries, and perivascular accumulation of inflammatory cells [29,30]. A further pathological study in dogs with chronic congestive heart failure (CHF) due to MMVD reported pulmonary arterial narrowing and intimal-medial thickening, supporting the concept that chronic LHD can induce structural changes in the arterial compartment [31]. Together, these findings support a continuum from venous congestion-dominant disease to a mixed phenotype with secondary arterial remodelling [4,8,32,33]. Clinicians estimate the echocardiographic probability of PH using TRV, integrated with supportive right-heart and PA indices, such as right-sided chamber enlargement, interventricular septal flattening, PA dilatation, and a shortened PA acceleration time, interpreted in the context of the underlying disease [4]. In chronic LHD, a shortened PA acceleration time and increased echocardiographic estimates of PVR may indicate the development of a superimposed pre-capillary component. However, these findings should be interpreted as evidence of secondary pulmonary vascular remodelling within group 2 PH, rather than as proof of primary PAH [8,33]. This distinction is important because canine PAH appears to exhibit a different pathological substrate, more closely resembling human PAH, with diffuse constrictive arteriopathy and, in severe cases, plexiform and dilatation lesions [34]. Determining whether PH is pre- or post-capillary is central to establishing aetiology, prognosis, and management in both species. Table 2 provides a crosswalk between haemodynamic definitions, echocardiographic features, and the corresponding clinical groups in human and veterinary classification systems [1,2,4].

## 3. Comparative Epidemiology

Pulmonary hypertension imposes a substantial global burden across the lifespan and is especially prevalent in older adults; best estimates suggest PH affects ~1% of the human population overall and approaches ~10% in people >65 years [35]. In the United Kingdom, observed prevalence has roughly doubled over the last decade and is currently ~125 cases per million, with referrals to specialist centres also rising [2]. Within the spectrum of PH, PAH is rare. The Global Burden of Disease 2021 analysis estimated ~192,000 people living with PAH worldwide in 2021 (age-standardised prevalence 2.28 per 100,000; ~62% female) [36]. In veterinary medicine, population-level prevalence is not established; however, echocardiographic evidence of PH is common among dogs with MMVD, affecting approximately 39% in an ACVIM B2/C cohort. Similarly, in West Highland White Terriers with canine idiopathic pulmonary fibrosis (CIPF), moderate-to-severe PH is present in about 60% at diagnosis, reflecting the predominance of left-sided cardiac and respiratory disease as major contributors in older canine populations [10,37]. Distributions among species are further shaped by regional factors: in humans, endemic infections like HIV and schistosomiasis significantly contribute to PAH in impacted areas [38,39]; high-altitude hypoxia causes high-altitude PH [40]; and there is mounting evidence that ambient air pollution is linked to the burden of pulmonary vascular disease and PH exacerbation [41]. In dogs, PH patterns and clinical identification are influenced by breed-linked respiratory disease (e.g., West Highland White Terriers with CIPF; brachycephalic obstructive airway syndrome (BOAS)), parasite endemicity (e.g., *Dirofilaria immitis*, *Angiostrongylus vasorum*), and severe pulmonary infections within the spectrum of respiratory disease and/or hypoxia-associated PH, including bacterial and aspiration pneumonia [37,42,43,44]. Overall, PH in both humans and dogs is most common in older individuals and is driven predominantly by LHD and respiratory disorders, although the relative contribution of infectious, environmental, and breed-related factors differs between species. Persistent data gaps in canine population estimates underscore the need for harmonised diagnostic criteria, such as the ACVIM probability-based approach, and multicentre registries spanning primary and referral settings [4].

## 4. Pathophysiology

Pulmonary hypertension is best understood not as a single disease entity, but as a final haemodynamic syndrome resulting from diverse underlying pathological processes [1,2]. Across PH phenotypes, coordinated abnormalities may develop within the endothelial, medial, and adventitial compartments of the pulmonary vasculature, with recurrent downstream features including vascular remodelling, endothelial dysfunction, altered vasoactive signalling, inflammation, thrombosis and, ultimately, RV maladaptation [28,45,46,47]. However, the relative importance, anatomical distribution, and temporal sequence of these processes differ markedly according to the underlying cause. For this reason, the pathobiology of PH is best interpreted according to phenotype rather than as a single uniform mechanism [1,4,28]. Table 3 summarises the principal mediators and pathways involved in PH, showing whether they are typically increased, decreased or dysregulated, together with their main biological effects and translational relevance [2,4,28,48,49].

In PAH, the primary lesion is centred in the small pre-capillary pulmonary arteries and arterioles [28]. A key early event is pulmonary arterial endothelial cell dysfunction, which disrupts barrier integrity, vasoactive balance, and crosstalk with pulmonary arterial smooth-muscle cells (PASMCs) and fibroblasts, thereby initiating a spectrum of vascular remodelling that ranges from medial hypertrophy and intimal fibrosis to angio-obliterative plexiform lesions [28,46,50]. Diverse, often synergistic triggers precipitate or amplify endothelial injury. Human PAH is characterised by endothelial injury and failed repair, dysregulated type II receptor for bone morphogenetic protein (BMP)/TGF-β signalling, reduced nitric oxide and prostacyclin activity, increased endothelin-1 signalling, proliferative smooth-muscle and fibroblast responses, immune activation and metabolic rewiring [28,40,45]. Genetic susceptibility has a major role in a subset of cases, particularly through rare variants affecting the BMP/TGF-β axis and allied pathways, including type II receptor for BMP, activin receptor type 1, mothers against decapentaplegic homologs, endoglin, growth differentiation factor 2, alongside genes involved in endothelial development and ion/signalling homeostasis such as SRY-box transcription factor 17, T-box transcription factor 4, potassium two-pore domain channel subfamily K, kinase insert domain receptor, caveolin 1, ATPase 13A3 and eukaryotic translation initiation factor 2 alpha kinase 4 [28,51]. These abnormalities bias signalling towards pro-proliferative, pro-fibrotic states and may increase susceptibility to environmental or acquired stressors such as hypoxia, viral infection, autoimmunity and drug or toxin exposure [28]. In parallel, matrix stiffening and disturbed shear stress activate mechanotransduction pathways, notably yes-associated protein/transcriptional co-activator with PDZ-binding motif signalling, which further promotes vascular-cell growth, migration and extracellular-matrix remodelling [46,52]. Pulmonary arterial endothelial cell dysfunction is also characterised by a vasoactive imbalance, with reduced nitric oxide and prostacyclin bioavailability and increased endothelin-1 signalling, thereby favouring vasoconstriction and muscularisation. In addition, platelet-derived growth factor and fibroblast growth factor 2 signalling, together with endothelial-to-mesenchymal transition (EndoMT), further reinforce intimal thickening and lesion complexity [28,50,53]. A pervasive immune–inflammatory component also sustains remodelling, with perivascular macrophages, lymphocytes, dendritic cells, and tertiary-lymphoid structures producing interleukin-6/interleukin-1–axis cytokines, chemokines, and growth factors that perpetuate endothelial injury and fibroblast activation [28]. Serotonergic signalling has also been implicated, as peripheral serotonin generated via tryptophan hydroxylase 1 (TPH1)-dependent synthesis promotes PASMC proliferation and inflammation in preclinical models; however, a recent phase 2b trial of the TPH1 inhibitor rodatristat ethyl did not meet its primary haemodynamic endpoint, underlining the need for caution when translating findings from experimental models to patients [54,55].

Across vascular cell types, metabolic rewiring sustains a proliferative and apoptosis-resistant phenotype, characterised by a shift toward aerobic glycolysis, augmented glutaminolysis, mitochondrial dysfunction with excess reactive oxygen species, and suppression of fatty-acid oxidation. These changes are now recognised in both pulmonary vasculature and the RV, providing targets for metabolic modulation [56,57]. Lesion biology in PAH spans medial hypertrophy, concentric laminar intimal fibrosis, and plexiform or angioproliferative lesions, the latter reflecting exuberant, clonally biassed endothelial proliferation with disorganised channels fed by bronchial/*vasa-vasorum* inputs in advanced disease. Together with vasoconstriction and thrombotic changes, these structural alterations raise PVR and mPAP [28]. A similar arteriopathic pattern has been described in canine idiopathic or primary PAH, supporting the comparative relevance of naturally occurring canine disease to pre-capillary vasculopathy [19,24,34].

In chronic thromboembolic pulmonary hypertension (CTEPH), unresolved organised thrombus within the proximal pulmonary vasculature coexists with a secondary distal microvasculopathy; loss of endothelial integrity and in situ thrombosis within small muscular arteries may further contribute to elevated pulmonary vascular resistance and persistent PH, including after pulmonary endarterectomy [58,59].

By contrast, PH associated with LHD develops as a consequence of chronically elevated left-sided filling pressures and pulmonary venous hypertension, rather than as a primary pulmonary arteriopathy [25,26]. In humans, the earliest lesions involve the venous and capillary compartments, although chronic congestion may progress to a mixed vasculopathy with secondary arterial remodelling, particularly in combined post- and pre-capillary PH [25,26,27]. The same concept is increasingly supported in dogs with MMVD, the most common cause of canine post-capillary PH [4,10]. In this setting, the initiating event is the backward transmission of pressure from the left atrium and pulmonary veins, followed by structural remodelling of the pulmonary arterial bed [4,25]. Histopathological studies in dogs with MMVD-associated PH demonstrate medial thickening, muscularisation of small pulmonary arteries and collagen deposition [29,31]. Perivascular inflammatory cells are also increased in this phenotype, with higher numbers of B lymphocytes, T lymphocytes, and macrophages surrounding remodelled pulmonary arteries [30]. Platelet biology appears relevant as well: dogs with MMVD and PH exhibit platelet hyperresponsiveness and increased platelet-neutrophil aggregate formation [60]. Preliminary evidence further suggests alterations in serotonin pathway signalling within pulmonary arteries and lung tissue in dogs with PH secondary to MMVD, although the mechanistic significance of these findings remains unclear [48,61,62]. Overall, in MMVD-associated PH, inflammation, platelet activation and other molecular abnormalities should be interpreted within a post-capillary, secondary remodelling framework, rather than extrapolated across all forms of canine PH [4,6,29].

In PH associated with fibrotic lung disease and/or hypoxia, the dominant mechanisms differ again [1,44]. In human fibrotic interstitial lung disease, and in West Highland White Terriers with CIPF, PH reflects a combination of destruction or rarefaction of the pulmonary vascular bed, hypoxic vasoconstriction, fibrosis-related vascular stiffening and secondary vascular remodelling [44,63,64,65]. The fibrotic lung is not merely a passive background lesion; rather, parenchymal and vascular injury evolve together [65]. In dogs with CIPF, the disease is associated with progressive collagen deposition, impaired gas exchange and emerging evidence of profibrotic macrophage programmes, making this phenotype mechanistically distinct from MMVD-associated PH [63,66,67]. Likewise, in brachycephalic obstructive airway syndrome, the relevant pathway is not venous congestion but chronic upper-airway obstruction with intermittent hypoxaemia, sleep-disordered breathing and large negative intrathoracic pressure swings, analogous in several respects to obstructive sleep apnoea in humans [43,68,69]. These group 3 phenotypes therefore differ fundamentally from PH associated with LHD in both trigger and tissue compartment, even though both may ultimately converge on increased pulmonary vascular resistance and RV afterload [1,25,44,63].

Importantly, parasitic PH has a species-specific expression. In dogs, heartworm disease (*Dirofilaria immitis*) is a major cause of pulmonary vascular injury and PH, via endothelial inflammation, proliferative endarteritis, thrombosis, and, in advanced cases, caval syndrome [42,70]. In humans, schistosomiasis is the principal parasitic disease associated with PAH, whereas human pulmonary dirofilariasis typically presents as a solitary pulmonary nodule (“coin lesion”) and does not cause PH [39,71].

Finally, venous and capillary disorders such as pulmonary veno-occlusive disease (PVOD) and pulmonary capillary haemangiomatosis (PCH) represent yet another distinct anatomical substrate. In these conditions, the dominant pathology involves venular occlusion, venous intimal fibrosis, capillary proliferation, interstitial oedema and secondary arterial changes. These disorders may mimic PAH haemodynamically, but differ fundamentally in lesion location and in their response to vasodilator therapy, with pulmonary oedema representing a recognised risk [72,73]. Naturally occurring canine PVOD/PCH-like syndromes are rare, but they are valuable from a comparative and One Health perspective because they reflect a venous/capillary pulmonary vascular phenotype that is not well represented by conventional arterial PH models [74,75,76].

**Figure 1:**
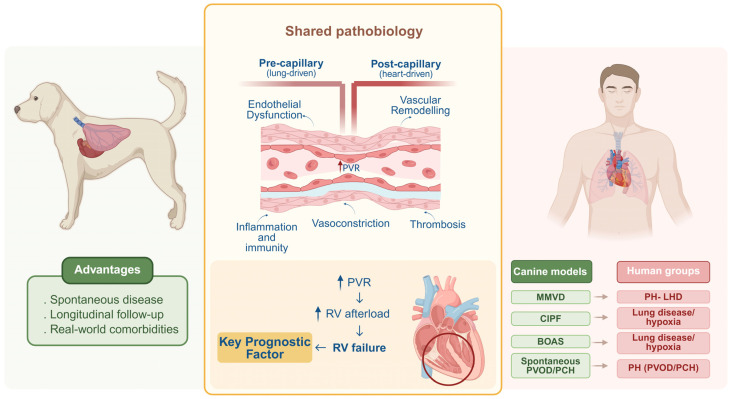
Bridging the translational gap in pulmonary hypertension: insights from spontaneous canine models. Conceptual overview of shared mechanisms across pulmonary hypertension phenotypes and the alignment of naturally occurring canine conditions with clinically relevant groups to support cross-species translation. BOAS: brachycephalic obstructive airway syndrome; CIPF: canine idiopathic pulmonary fibrosis; LHD: left-heart disease; MMVD: myxomatous mitral valve disease; PCH: pulmonary capillary haemangiomatosis; PH: pulmonary hypertension; PVOD: pulmonary veno-occlusive disease; PVR: pulmonary vascular resistance; RV: right ventricle. Created in BioRender. Reis, A. (2026) https://BioRender.com/mblw2ql.

Across all phenotypes, persistent elevation of pulmonary vascular load ultimately challenges the RV. Early adaptation involves increased contractility and concentric hypertrophy, thereby preserving right ventricle-pulmonary artery coupling despite rising afterload [1,47]. With sustained pressure overload, however, this adaptive state may progress to maladaptation, characterised by chamber dilatation, increased wall stress, capillary rarefaction with relative ischaemia, fibrosis, maladaptive inflammation and broader metabolic remodelling, including a shift from fatty acid oxidation towards glycolysis [47,56,57]. Progressive right ventricle-pulmonary artery uncoupling heralds clinical right-sided heart failure and is a major determinant of prognosis across PH aetiologies [47]. Thus, although the final common pathway of increased RV load is shared across human and canine PH, the route by which it is reached differs substantially between pre-capillary arteriopathy, post-capillary venous hypertension, fibrotic-hypoxic disease, chronic thromboembolic disease, parasite-associated vascular injury and venous/capillary pulmonary vascular disorders (Figure 1). Recognising these mechanistic differences is essential for accurate comparative interpretation and for the development of phenotype-appropriate biomarkers and therapies [1,4].

## 5. Clinical Presentation

In humans, PH most often presents with exertional dyspnoea, fatigue, and reduced exercise tolerance, reflecting rising RV afterload and impaired RV–PA coupling. Exertional presyncope/syncope is a red flag for advanced haemodynamic limitation [1,2,77]. With progression, right-sided heart failure manifests as peripheral oedema, abdominal distension/ascites, early satiety/weight gain, elevated jugular venous pressure, and hepatomegaly, while low forward output contributes to dizziness, cool extremities, and delayed capillary refill. Palpitations (often due to atrial arrhythmias) are common; bendopnoea, haemoptysis, and cyanosis occur in advanced disease. Consequences of PA dilatation are uncommon but characteristic: exertional angina from left main coronary artery compression, dysphonia/hoarseness from left recurrent laryngeal nerve palsy (Ortner’s syndrome), and cough/wheeze or recurrent infection from bronchial compression. On examination, clinicians may detect a loud/accentuated P2, an RV S3, a holosystolic tricuspid regurgitation murmur, and, when severe, a diastolic pulmonary regurgitation murmur; an RV heave and fixed splitting of S2 may also be present [1,2]. In Group 2 and Group 3 PH, symptoms/signs attributable to left-sided/valvular disease or parenchymal lung disease/hypoxaemia commonly coexist and should be integrated into diagnostic probability and downstream testing [1,2,77].

In dogs, clinical signs are likewise non-specific and derive from impaired cardiopulmonary reserve and RV dysfunction. Typical presentations include exercise intolerance, tachypnoea/dyspnoea at rest or with minimal exertion, syncope/collapse (often exertional or excitement-triggered), and cyanotic or pale mucous membranes; cough, lethargy and episodic respiratory distress frequently reflect the underlying aetiology (e.g., MMVD, interstitial lung disease (ILD,) BOAS, or parasitic disease) rather than PH per se [4,6,78,79]. Physical examination may show signs of right-sided congestive failure such as abdominal distension/ascites, jugular venous distension with or without positive hepatojugular reflux, and right-heart murmurs (tricuspid regurgitation most commonly); in advanced cases, a diastolic murmur of pulmonary regurgitation and a loud/split second heart sound have been described [4,80]. Respiratory pattern and auscultatory findings (crackles, wheeze, increased effort) often point to concurrent lung disease or post-capillary pulmonary congestion (e.g., MMVD). Given that clinical signs are often insensitive and non-specific, echocardiographic probability assessment using TRV together with supportive right-heart and PA indices is recommended to estimate the likelihood of PH and direct further investigation and management [4].

## 6. Diagnosis

In humans, the initial assessment of exertional dyspnoea and suspected PH begins with history, examination (including pulse oximetry), resting electrocardiogram, and natriuretic peptides (B-type natriuretic peptide (BNP)/N-terminal pro-B-type natriuretic peptide (NT-proBNP)) to assess RV strain. Transthoracic echocardiography is the first-line non-invasive test to estimate the probability of PH and to identify alternative/causal cardiac disease; contemporary frameworks emphasise integrating TRV with supportive right-heart and PA indices rather than relying on Doppler alone [1,2]. When parenchymal lung disease is possible, spirometry with lung volumes, diffusing capacity of the lungs for carbon monoxide, arterial blood gases, chest radiography, and high-resolution computed tomography (HRCT) are recommended; cardiopulmonary exercise testing can clarify mechanisms of exercise limitation and unmask pulmonary vascular limitation [1,2,81]. Screening for CTEPH relies on ventilation/perfusion scintigraphy (preferred initial test); abnormal scans prompt computed tomography (CT) pulmonary angiography and/or selective angiography at an expert centre to define operability and distal disease [59,82]. Referral to a PH centre is advised for intermediate/high echocardiographic probability, the presence of PAH risk factors, prior pulmonary embolism with CTEPH suspicion, or any red flags (rapid symptom progression, exertional syncope, evident RV dysfunction, rising biomarkers, low-output signs, poorly tolerated arrhythmias, or haemodynamic instability) [2]. The gold standard for diagnosis and haemodynamic classification is RHC with direct pressure measurements and calculation of PVR to distinguish post-capillary (Group 2) from pre-capillary forms (Groups 1, 3, 4, 5); acute vasoreactivity testing is reserved for idiopathic/heritable/drug-induced PAH to identify calcium-channel-blocker responders [1,2]. Additional aetiologic work-up should be guided by the clinical phenotype together with biochemical and imaging findings that suggest specific underlying causes of pulmonary hypertension. Accordingly, targeted investigations may include autoimmune serologies when connective tissue disease is suspected, thyroid and HIV testing, abdominal imaging when portal hypertension is considered, and ventilation/perfusion scanning when chronic thromboembolic disease is a possibility; cardiac magnetic resonance imaging further refines right-ventricular size and function, pulmonary artery morphology and flow, provides prognostic information, and is particularly useful when echocardiography is inconclusive or technically limited [1,83].

In dogs, PH may occur in isolation or as a complication of LHD, respiratory/hypoxic disease, thrombo-embolic processes, or parasitic infection. While RHC remains the definitive haemodynamic test, its invasiveness limits routine use; thus, diagnosis follows an echocardiographic probability approach anchored in TRV and corroborating signs of right-sided pressure/volume overload and PA changes (e.g., RV/RA enlargement, interventricular septal flattening, PA dilatation, shortened PA acceleration time/acceleration time to ejection time ratio) [4,5]. Probability categories commonly consider TRV > 3.4 m/s as high, 3.0–3.4 m/s as intermediate when accompanied by supportive findings, and acknowledge that TRV < 3.0 m/s does not exclude PH when right-sided indices are compelling [4]. Ancillary testing, such as thoracic radiography, electrocardiogram, pulse oximetry or arterial blood gas, and measurement of NT-proBNP and cardiac troponin I as supportive biomarkers aid in assessing severity and comorbidities, while advanced imaging is reserved for suspected thromboembolic or ILD [4]. Echocardiographic estimates should be interpreted in context, because Doppler gradients correlate only moderately with catheter values and perform best as part of an integrated probability model. Emerging indices, such as right PA distensibility assessed by echocardiography and the pulmonary trunk-to-aorta ratio measured by CT in selected phenotypes, may also aid serial assessment [4,84].

Across species, human RHC-based haemodynamic standards and veterinary echocardiographic probability frameworks are complementary. Aligning their thresholds and supportive indices (TRV, RV size/function metrics, PA indices, biomarkers) enables comparable phenotyping, improves triage to expert centres in humans or to advanced diagnostic testing in dogs, and strengthens translational studies addressing shared aetiologies such as PH-LHD and fibrotic-lung-disease PH.

## 7. Treatment

In PH, treatment is phenotype-specific, and in both species the therapies currently available are more reliable at improving haemodynamic and clinical status than at reversing established pulmonary vascular remodelling. In human medicine, current treatment of PAH follows a risk-stratified approach targeting the endothelin, nitric-oxide–cyclic GMP, prostacyclin, and activin pathways. Calcium-channel blockers remain appropriate only for the small subset of patients who demonstrate acute vasoreactivity [2,85]. More recently, sotatercept has expanded the therapeutic landscape as an add-on therapy that modulates dysregulated activin/TGF-β signalling, with demonstrated benefit across haemodynamic, functional, and clinical end-points [49,86]. Management of non-PAH phenotypes is determined primarily by the underlying disease and by timely referral into specialist care pathways. In PH associated with ILD, inhaled treprostinil currently provides the strongest randomised-trial evidence [2,87,88]. In CTEPH, optimal treatment requires expert-centre assessment for pulmonary endarterectomy, balloon pulmonary angioplasty, and long-term anticoagulation, with riociguat reserved for patients with inoperable disease or persistent/recurrent PH after intervention [2,59]. By contrast, no PH-specific therapy has yet shown convincing disease-modifying benefit in PH due to LHD, where management continues to focus on optimisation of the primary cardiac condition [89].

In dogs, the therapeutic evidence base is far less developed, and treatment remains guided largely by clinical phenotype, comorbidities, and pragmatic extrapolation. Sildenafil is the most widely used PH-specific agent, with tadalafil providing a practical longer-acting alternative in selected cases [79,90]. Adjunctive therapy should be directed at the underlying disorder and may include oxygen supplementation, antithrombotic treatment for thromboembolic disease, diuretics and cardiac standard therapy for left-sided CHF (including pimobendan when appropriate to the cardiac phenotype), antiparasitic treatment for heartworm disease or angiostrongylosis, and medical or surgical management of upper-airway obstruction in brachycephalic dogs. Importantly, pulmonary vasodilators must be used cautiously in dogs with LHD, as excessive reduction in pulmonary vascular tone in the presence of persistently elevated left-sided filling pressures may precipitate pulmonary oedema [4]. Interest in additional veterinary agents is growing, although current evidence remains preliminary. Small case series suggest potential benefit from ambrisentan in sildenafil-refractory dogs, and exploratory studies of prostacyclin-pathway modulation have reported favourable haemodynamic effects; however, neither approach is supported by robust phenotype-specific clinical trials, and neither can currently be considered established therapy [91,92,93]. From a translational perspective, the disparity between the mature human therapeutic landscape and the more limited canine evidence base should be viewed as informative rather than restrictive. Human PH research provides mechanistic and trial-design templates, whereas naturally occurring canine disease offers a comparative setting in which imaging end-points, biomarker trajectories, tolerability, and treatment response can be evaluated under real-world comorbidity conditions that are seldom reproduced in reductionist laboratory models [4,13,85].

## 8. The Dog as a Spontaneous Model of Pulmonary Hypertension

As outlined above, several canine disorders recapitulate essential mechanisms underlying human PH. Here, we focus on a selected group of these naturally occurring conditions and outline their relevance within a One Health comparative medicine setting.

### 8.1. Pulmonary Veno-Occlusive Disease and Pulmonary Capillary Hemangiomatosis

Pulmonary veno-occlusive disease and PCH are rare angio-obliterative causes of pre-capillary PH in humans. Pulmonary veno-occlusive disease predominantly targets post-capillary venules/small pulmonary veins, while PCH features proliferation of pulmonary capillaries within alveolar septa. Clinically and radiologically, they overlap and are often considered ends of a shared spectrum within Group 1 pulmonary vascular disease [73,94]. Spontaneous canine cases, though rare, are increasingly recognised and provide a natural-disease model that captures key human features within complex comorbidity settings [72,74,75].

In humans, PVOD/PCH spans infancy to late adulthood, with clustering in young to middle-aged adults; profound resting/exertional hypoxaemia, disproportionately low diffusing capacity of the lungs for carbon monoxide, and rapid functional decline are typical [73,94]. In dogs, reported cohorts are older (median ~10–11 years), of various breeds and sexes, and present with progressive dyspnoea, exercise intolerance, cyanosis/hypoxaemia, and signs of right-sided failure; cough, collapse, and lethargy are frequent but non-specific [72,74,75]. Diagnostic confusion with LHD, ILD, or idiopathic PAH is common in both species [76]. Human HRCT shows a suggestive triad—centrilobular ground-glass opacities, smooth interlobular septal lines, and mediastinal lymph-node enlargement—with frequent occult alveolar haemorrhage; ventilation/perfusion scans are usually normal or non-segmentally abnormal (unlike mismatched defects in CTEPH) [73]. In dogs, thoracic CT and radiography can show diffuse interstitial/ground-glass change and PA enlargement; echocardiography documents pre-capillary PH physiology (elevated TRV with right-sided remodelling), while left-sided disease is absent or insufficient to explain the severity of PH, an important diagnostic cue for PVOD/PCH suspicion [74,75]. Because surgical lung biopsy carries high risk in both species, diagnosis relies on clinic-radiological probability, with histology reserved only for selected cases or *post-mortem* confirmation [73,75]. Human PVOD exhibits fibrotic intimal thickening/occlusion of small pulmonary veins and venules, arterialisation of venules, and lymphatic remodelling. Pulmonary capillary hemangiomatosis shows diffuse capillary proliferation with alveolar septal expansion, often co-existing with venular lesions reinforcing a *continuum* concept. Biallelic EIF2AK4 mutations are pathognomonic for heritable PVOD/PCH and allow a non-invasive genetic diagnosis in appropriate clinical contexts. By contrast, type II receptor of BMP is linked to PAH rather than PVOD [73,94]. Canine reports describe PVOD-like and PCH-like histology closely mirroring human lesions [95], but canine genetics remain undefined; targeted screening in a single dog did not identify known human PVOD variants [74].

In humans, PAH-targeted vasodilators can precipitate pulmonary oedema because downstream venous obstruction limits pulmonary venous outflow. Although carefully titrated use may be attempted in selected cases for symptomatic relief, lung transplantation remains the only definitive therapy, and outcomes without transplant are poor, with historical median survival of approximately two years. Supportive management includes oxygen supplementation, diuretics, and judicious anticoagulation, with bleeding risk carefully balanced [73,94]. In dogs, evidence is limited to small case series. Management is predominantly supportive—usually oxygen therapy and diuretics—and extreme caution is required if vasodilators are trialled. Prognosis is generally guarded to poor [74,75,95]. These shared therapeutic challenges highlight the value of naturally occurring canine disease for investigating non-invasive diagnostic approaches, imaging biomarkers, and safety signals for vasoactive therapies.

Spontaneous canine PVOD/PCH offers an ethically attractive and translationally relevant platform for (i) evaluating clinic-radiologic diagnostic algorithms (including HRCT signatures, echocardiographic phenotypes, and surrogates of diffusing capacity where feasible), (ii) validating biomarkers of venous/capillary injury, and (iii) exploring genetic architecture, such as analysis of the EIF2AK4 orthologue, under the pressures of naturally occurring disease [73,94]. Greater clinical awareness is warranted in dogs presenting with severe pre-capillary PH, marked hypoxaemia, disproportionately low diffusing capacity surrogates, characteristic HRCT triad features, and no sufficient LHD, particularly when pulmonary oedema arises following initiation of PAH-target therapy [72,75].

### 8.2. Left-Heart Disease—Myxomatous Mitral Valve Disease

Left heart disease is the most common cause of PH in both species, and MMVD is the dominant LHD phenotype in dogs, offering a natural model for post-capillary PH and for the post- to pre-capillary *continuum* recognised in human PH-LHD [4,25]. Small-breed, ageing dogs with MMVD mirror key clinical, imaging, and biomarker trajectories seen in human mitral valve prolapse (MVP) with regurgitation, while also enabling evaluation of pragmatic medical and emerging interventional strategies [96,97].

Canine MMVD is highly prevalent in small breeds (e.g., Cavalier King Charles Spaniels), is age-associated, and follows a predictable course from leaflet thickening to clinically important mitral regurgitation (MR) and, in a subset, left-sided CHF [96]. In humans, the closest analogue is MVP with MR; prevalence is ~2–3% and increases with age, often diagnosed in mid-to-late adulthood and enriched in connective-tissue disorders (e.g., Marfan, Ehlers–Danlos) [97,98]. Surgical mitral repair/replacement is routine for severe MR in humans, whereas dogs are typically managed medically, though early veterinary experience with transcatheter edge-to-edge repair is emerging [96,99,100,101]. Echocardiography is the diagnostic cornerstone in both species, while thoracic radiography is widely used in dogs to assess cardiomegaly and pulmonary congestion, and circulating NT-proBNP is increasingly adopted for disease staging and prognosis [96,102].

Normal mitral valves in both species comprise layered atrialis–spongiosa–fibrosa–ventricularis architecture. In MMVD, this is disrupted by proteoglycan expansion of the spongiosa, collagen/elastin disorganisation, and chordal/leaflet deformity [103,104]. Human MMVD exhibits phenotypes from fibro-elastic deficiency to Barlow’s disease; canine MMVD most closely resembles the latter (diffuse myxoid change), typically involving both leaflets [104,105]. Biomechanically, increased extensibility and reduced tensile strength promote prolapse and regurgitation. Key convergent processes include valvular interstitial cell activation with α-SMA expression, EndoMT, and TGF-β pathway up-regulation that drives extracellular matrix remodelling [103,104,105]. A conserved serotonin (5-HT) axis interacts with TGF-β/Extracellular signal-regulated kinase signalling: in canine MMVD-PH, lung and PA tissues show altered TPH1/5-HT receptor expression and context-dependent serotonin transporter behaviour, correlating with echocardiographic indices of pressure load/remodelling; in humans, receptor usage varies by dataset (e.g., 5-HTR2B vs 5-HTR2A) [48,61,62,104,106]. Extracellular matrix programmes diverge subtly: human MMVD more often shows broad collagen gene up-regulation (COL1A1/COL3A1) and matrix metalloproteinase (MMP) activation, whereas dogs demonstrate prominent versican accumulation and distinct MMP signatures (e.g., ↑MMP-12) with comparatively limited fibrosis [103,104,105]. MicroRNA signals (e.g., miR-29, miR-132, let-7c) modulate extracellular matrix/valvular interstitial cell behaviour in dogs, with human datasets supporting analogous regulatory themes [105]. When PH complicates canine MMVD, lungs exhibit medial thickening, smooth-muscle cell (SMC) hyperplasia/hypertrophy, arteriolar muscularisation, and collagen deposition, with perivascular lymphocytes/macrophages; indices of apoptosis favour SMC survival (↓Bax, ↓caspase-3/-8; ↑Bcl-2) [30,48,107]. Mast-cell abundance appears stage-dependent (higher in MMVD without PH) [30]. These findings mirror human PH-LHD, in which venous hypertension, hypoxic vasoconstriction, inflammation, and serotonergic signalling co-evolve to raise PVR and RV load [108,109].

Collectively, canine MMVD constitutes a robust, naturally occurring translational model of human degenerative mitral valve disease. Its breed-linked susceptibility, predictable longitudinal course, and close histopathological concordance with human MVP/MR create a practical platform for preclinical discovery and therapeutic evaluation. At a mechanistic level, both species exhibit conserved programmes—valvular interstitial cell activation, EndoMT, extracellular-matrix re-patterning, and dysregulated TGF-β/serotonin signalling—while interspecies differences in fibrotic tone and matrix composition provide informative contrast rather than contradiction [96,103,104,105,106]. These parallels enable cross-species endpoints (advanced echocardiography, circulating biomarkers, and tissue/omics readouts) and support the dog as a clinically and mechanistically relevant model to probe ventricular–valvular coupling, refine risk stratification, and test medical and interventional strategies, including emerging transcatheter repair, under real-world comorbidities and lifespans [96,99,100,102].

### 8.3. Hypoxia and/or Lung Diseases

#### 8.3.1. Idiopathic Pulmonary Interstitial Fibrosis of the West Highland White Terrier

Idiopathic pulmonary fibrosis (IPF) in humans and CIPF in West Highland White Terriers are chronic, progressive ILDs with poor prognosis and frequent pre-capillary PH. West Highland White Terriers show breed-linked susceptibility (middle-aged to older), whereas human IPF is male-predominant and typically presents in late adulthood [63,110].

Across species, onset is insidious with exertional dyspnoea and a usually dry cough that progress to tachypnoea, reduced exercise tolerance, and hypoxaemia; on auscultation, fine “Velcro-type” crackles are common and, in humans, predict fibrotic ILD patterns on imaging [65,111,112]. In dogs, signs evolve from chronic cough and exercise intolerance to increased respiratory effort and dyspnoea at rest or with minimal exertion; advanced cases may develop cyanosis or exercise-induced collapse. When pre-capillary PH complicates CIPF, exertional syncope or weakness may occur and, if right-sided failure develops, jugular venous distension and ascites can be observed [63,111,113]. Arterial blood gas often documents resting or exertional hypoxaemia, and the 6 min walk test serves as a pragmatic functional endpoint in both species [64,65]. In human IPF, HRCT is the cornerstone of diagnosis, with surgical lung biopsy reserved for indeterminate patterns in a multidisciplinary framework; the same HRCT-first logic increasingly informs veterinary practice when available [65]. CT-angiography in West Highland White Terriers may also identify pre-capillary PH risk markers and furnish non-invasive vascular endpoints for longitudinal monitoring [64]. Routine thoracic radiography supports staging/exclusion of differentials, while reliable serum biomarkers with diagnostic or prognostic utility remain lacking in both species [63,65].

In both species, repetitive alveolar epithelial injury and aberrant repair drive myofibroblast accumulation and collagen-rich matrix deposition that distorts lung architecture. Human disease is characterised by fibroblast foci and marked spatial-temporal heterogeneity, consistent with a usual interstitial pneumonia pattern, whereas dogs exhibit interstitially scattered myofibroblasts with broadly similar patterns of matrix remodelling [63,110]. TGF-β signalling is implicated across species: canine studies document pathway activation and altered storage/activation of TGF-β1 with downstream regulatory changes in CIPF, and comparative work supports shared molecular networks with human IPF [66]. Putative epidemiological contributors (e.g., cigarette smoke, gastro-oesophageal reflux, viral exposures) are better defined in humans, and canine studies to date have not supported a herpesvirus association with CIPF [63,110]. From a genetic standpoint, human IPF risk reflects a polygenic architecture with common and rare variants (including mucin, telomere-maintenance, and surfactant-pathway genes), while in dogs the strong breed predisposition (West Highland White Terriers) indicates heritable susceptibility yet no single causal locus has been consistently identified; taken together, this suggests overlapping pathogenic programmes with species-specific genetic backgrounds modulating penetrance and expression [63,66,110,113,114].

Canine idiopathic pulmonary fibrosis in West Highland White Terriers represents a high-face-validity model of human IPF, enabling investigation of epithelial–mesenchymal crosstalk, matrix biology, the progression to pre-capillary PH, and the development of pragmatic, cross-species monitoring endpoints [63]. Although the upstream triggers remain incompletely defined in both species, the convergence in clinical phenotype, gas-exchange impairment, and progressive fibrotic remodelling supports CIPF as a One Health platform to advance non-invasive diagnostics, such as HRCT pattern recognition, functional walk tests, arterial blood gas profiling, and exploratory circulating or exhaled biomarkers, refine risk stratification, and pilot antifibrotic and PH-directed strategies under real-world comorbidities and longitudinal follow-up [63,64,65,111,112,113].

#### 8.3.2. Brachycephalic Obstructive Airway Syndrome

Within One Health/comparative frameworks, BOAS aligns with Group 3 PH (lung disease and/or hypoxia). Many brachycephalic dogs develop sleep-disordered breathing (SDB) superimposed on fixed and dynamic upper-airway obstruction, providing a spontaneous analogue of human obstructive sleep apnoea (OSA). Shared biology—recurrent hypoxia–reoxygenation, oxidative stress, endothelial dysfunction, inflammation, and pro-thrombotic shifts—can increase PVR and impose right-ventricular load [2,4,68,69,115,116,117].

Human OSA is characterised by snoring, witnessed apnoea/hypopnoea, non-restorative sleep, and daytime sleepiness due to sleep-related pharyngeal collapse with cyclical desaturations; overnight polysomnography is the diagnostic standard [69,115]. In brachycephalic dogs (e.g., Bulldogs, Pugs, French Bulldogs), BOAS presents with stertor/stridor, exercise and heat intolerance, sleep disruption, cyanotic episodes, and—when severe—syncope/collapse; English Bulldogs show sleep-stage–linked desaturation patterns analogous to human OSA, underscoring face validity as a natural model [68,118]. Risk is modified by age and obesity in both species; in dogs, BOAS severity is an independent SDB risk factor [117,118]. Veterinary assessment relies on structured BOAS grading, exercise/heat-challenge testing, and whole-body barometric plethysmography (WBBP) to quantify obstruction in unsedated dogs; cross-sectional imaging and dynamic airway endoscopy delineate multilevel lesions (stenotic nares, elongated/thickened soft palate, laryngeal collapse, and possibly hypoplastic trachea). Airway surgery improves signs, but residual SDB/hypoxaemia may persist, supporting longitudinal physiological monitoring (e.g., WBBP, oximetry surrogates) [117,119].

Repeated intermittent hypoxia associated with sleep-disordered breathing in BOAS promotes oxidative stress, sympathetic activation, endothelial dysfunction and inflammation, favouring pulmonary vasoconstriction and vascular remodelling; accordingly, OSA-related hypoxia is recognised within Group 3 PH mechanisms [2,69]. BOAS has also been associated with hypercoagulability and platelet activation, which may further increase pulmonary vascular load [120,121]. In most cases, BOAS-related pathophysiology results in mild-to-moderate Group 3 PH, particularly when concurrent lower-airway disease is present [4,117].

Brachycephalic dogs provide a naturally occurring and ethically attractive model for studying OSA-related intermittent hypoxia and its pulmonary vascular consequences. This model enables evaluation of non-invasive monitoring endpoints (such as WBBP metrics and oximetry-based surrogates) and assessment of the effects of airway surgery, weight management, and oxygen or positive-pressure support, where appropriate, on symptoms, gas exchange, coagulation, and pulmonary haemodynamics under real-world comorbidities and lifespans.

## 9. Implications for One Health

A One Health perspective is particularly relevant to PH, a syndrome that is biologically heterogeneous, clinically chronic, and strongly shaped by comorbidities [1,4,28]. Although naturally occurring canine disease does not replicate human PH in its entirety, several major phenotypes are recapitulated with a degree of anatomical, temporal, and clinical realism that is difficult to achieve in experimental models [1,4,19,28]. Within this context, selected canine conditions provide valuable comparative insight. Pulmonary hypertension secondary to LHD, particularly MMVD, offers a useful model of post-capillary PH and secondary pulmonary vascular remodelling [1,4,8]. Fibrotic interstitial lung disease in West Highland White Terriers and BOAS-associated hypoventilation and hypoxaemia in brachycephalic breeds, parallel key hypoxia- and lung disease-driven mechanisms. Likewise, specific venous and capillary disorders may help illuminate rarer PH phenotypes [1,4,37,75,111,117]. The value of this comparative perspective lies not in assuming equivalence between species, but in using each to clarify the other. A One Health approach can support harmonised phenotyping, shared imaging and biomarker endpoints, and the development of prospective registries and biobanks that enable molecular and clinical data to be analysed in parallel [1,122]. Equally importantly, it can help delineate points of divergence between species, reducing the risk of false equivalence and strengthening comparative interpretation.

## 10. Future Perspectives

Several priorities should shape future research in PH. First, the initiation and progression of PH must be defined more precisely. This includes clarifying the interactions among genetic susceptibility, environmental exposures, including hypoxia and pollution, infectious and immune-mediated drivers, and the critical point at which pulmonary vascular changes become functionally irreversible. Cross-species studies capable of detecting the earliest measurable abnormalities, including signals of endothelial injury, RV–pulmonary artery uncoupling, and microvascular rarefaction, will be important for shifting investigation and intervention earlier in the disease course. Second, the molecular and cellular hierarchy sustaining PH requires deeper resolution. Determining which cell populations are necessary and sufficient in distinct aetiologies, and whether conserved hierarchies exist across PH groups, will help prioritise therapeutic targets. Comparative single-cell and spatial multi-omics analyses of canine and human tissues may help identify shared signalling nodes, including TGF-β, endothelin, serotonin, and inflammatory pathways, while also revealing species-specific modifiers that can inform model selection and trial design [28,123]. Third, treatment strategies must evolve beyond vasodilation alone towards disease-modifying approaches capable of halting or reversing remodelling, not only in group 1 PAH but also in PH associated with LHD and lung disease [1,4,13,124]. Mechanism-based platform trials integrating anti-inflammatory, anti-fibrotic, metabolic, and RV-directed approaches alongside current standards of care are, therefore, warranted [85,124,125]. Naturally occurring canine PH may offer a pragmatic setting in which such candidates can be evaluated under authentic conditions of comorbidity, with repeated imaging, serial biomarker sampling, and clinically relevant outcomes. A fourth priority is personalisation and broader inclusion. Biomarker-guided phenotyping, imaging signatures, and integrated genetic and exposomic profiling should help refine treatment intensity and therapeutic choice [123,124]. This is likely to be particularly relevant in patients with mildly abnormal haemodynamic, in whom closer monitoring, refined phenotyping, and prospective evaluation of earlier intervention may be especially relevant, as well as in populations historically underrepresented in clinical trials [1,2,125]. In dogs, breed-associated risks and relatively predictable disease trajectories may also provide a useful framework for the development and validation of stratification tools. Supportive and non-pharmacological strategies likewise deserve rigorous evaluation. These include structured exercise and rehabilitation, nutritional optimisation, oxygen or ventilatory support (including targeted BOAS management and weight reduction in dogs), and interventional approaches such as CTEPH management in humans and evolving structural cardiology options in veterinary practice [1,4,59,117]. Finally, durable progress will depend on infrastructure. Harmonised, prospective registries and biobanks; common data elements across species (clinical class, echocardiographic probability, RV function indices, HRCT parenchymal and vascular scores); and shared endpoints (exercise capacity analogues, natriuretic peptides, survival, RV remodelling metrics) will be essential to support rigorous cross-species comparative research.

## 11. Conclusions

Pulmonary hypertension remains a complex and progressive syndrome in which shared biological mechanisms coexist with substantial clinical and mechanistic heterogeneity. Although major advances have been made in understanding pathobiology and improving clinical management, PH remains characterised by marked heterogeneity, delayed recognition, incomplete risk stratification, and limited capacity to reverse established vascular remodelling. Taken together, the available evidence supports naturally occurring canine disease as a relevant comparative model that can complement experimental systems and strengthen translational investigation within a rigorous One Health context. Future work should focus on earlier disease detection, improved mechanistic stratification, development of disease-modifying therapies, and the establishment of harmonised comparative research platforms to advance PH care in both humans and dogs.

## Figures and Tables

**Table 1:** Comparative clinical classification of pulmonary hypertension in humans and dogs.

Category	HUMANS	DOGS
**Group 1**	**PAH**	
	Idiopathic (long-term responders to calcium channel blockers); heritable; associated with drugs and toxins, connective tissue disease, HIV infection, portal hypertension, congenital heart disease, or schistosomiasis; PAH with features of venous/capillary (PVOD/PCH) involvement; persistent PH of the newborn	Idiopathic; heritable; associated with drugs and toxins, congenital cardiac shunts, pulmonary vasculitis, or pulmonary vascular amyloid deposition
**Group 1’**	Included in Group 1	**PH due to PVOD or PCH**
**Group 2**	**PH associated with LHD**	
	Heart failure with preserved, mildly reduced, or reduced ejection fraction; cardiomyopathies with specific aetiologies; valvular heart disease (aortic, mitral or mixed valvular disease); congenital or acquired cardiovascular conditions leading to post-capillary PH	Left ventricular dysfunction (dilated cardiomyopathy, myocarditis); acquired valvular disease (MMVD, valvular endocarditis); congenital or acquired left-heart inflow/outflow tract obstruction and congenital cardiomyopathies (mitral valve dysplasia, mitral stenosis, aortic stenosis)
**Group 3**	**PH associated with lung diseases and/or hypoxia**	
	COPD/emphysema; ILD; combined pulmonary fibrosis and emphysema; other parenchymal lung diseases; non-parenchymal restrictive disease (hypoventilation syndromes and pneumonectomy); hypoxia without lung disease (e.g., high altitude); developmental lung disorders	Chronic obstructive airway disorders (tracheal or mainstem bronchial collapse, bronchomalacia); primary pulmonary parenchymal disease (ILD, infectious pneumonia and diffuse pulmonary neoplasia); obstructive sleep apnoea/sleep disordered breathing; chronic high-altitude exposure; developmental lung disease; miscellaneous (bronchiolar disorders, bronchiectasis, emphysema, pneumonectomy)
**Group 4**	**PH associated with pulmonary artery obstructions**	
	Chronic thromboembolic PH; other PA obstructions	Acute PE/PT/PTE (massive PE/PT/PTE with RV dysfunction or submassive PE/PT/PTE without RV dysfunction); chronic PE/PT/PTE
**Multifactorial or unclear mechanisms**	**Group 5:** Haematological disorders; systemic disorders (sarcoidosis, pulmonary Langerhans cell histiocytosis, neurofibromatosis type 1); metabolic disorders; chronic renal failure with or without haemodialysis; pulmonary tumour thrombotic microangiopathy; fibrosing mediastinitis; complex congenital heart disease	**Group 6:** Disorders having clear evidence of two or more underlying groups 1–5 pathologies contributing to PH; masses compressing the pulmonary arteries (e.g., neoplasia, fungal granuloma, etc.); other disorders with unclear mechanisms
**Veterinary-specific**		**Group 5:** PH associated with parasitic disease (*Dirofilaria immitis* or *Angiostrongylus* infection)

COPD: chronic obstructive pulmonary disease; HIV: human immunodeficiency virus; ILD: interstitial lung disease; LHD: Left-heart disease; OSA: obstructive sleep apnoea; PA: pulmonary artery; PAH: pulmonary arterial hypertension; PCH: pulmonary capillary haemangiomatosis; PE: pulmonary embolism; PH: pulmonary hypertension; PT: pulmonary thrombosis; PTE: pulmonary thromboembolism; PVOD: pulmonary veno-occlusive disease; RV: right ventricle. Adapted from current human PH classification frameworks and the ACVIM consensus statement [1,2,4].

**Table 2:** Comparative overview of pre- and post-capillary pulmonary hypertension: haemodynamic definitions, echocardiographic indicators, and group mapping.

	PRE-CAPILLARY PH	POST-CAPILLARY PH
**Haemodynamic** **definition by RHC (human criteria)**	• mPAP > 20 mmHg • PAWP ≤ 15 mmHg • PVR > 2 WU	mPAP > 20 mmHg, PAWP > 15 mmHg, subdivided as: • Isolated post-capillary: PVR ≤ 2 WU • Combined post- and pre-capillary: PVR > 2 WU
**Echocardiographic** **supportive findings**	• Elevated TRV (context-dependent) • Enlargement RV/RA, RV hypertrophy, septal flattening (D-shape) • PA dilatation, short PA acceleration time/low AT:ET • LA size often normal; no primary mitral pathology	• LA enlargement, mitral valve disease or other LHD features • Doppler patterns consistent with elevated LAP (e.g., transmitral E/A, ↑E/E′) • Pulmonary venous congestion patterns; elevated TRV in proportion to LAP
**Clinical PH groups in dogs**	Group 1—PAH Group 3—Respiratory disease/hypoxia Group 4—PA obstruction Group 5—Parasitic disease Group 6—Multifactorial and/or unclear mechanisms	Group 2—LHD Group 6—Multifactorial and/or unclear mechanisms
**Clinical PH groups in humans**	Group 1—PAH Group 3—Lung disease and/or hypoxia Group 4—PA obstructions Group 5—Multifactorial and/or unclear mechanisms	Group 2—LHD Group 5—Multifactorial and/or unclear mechanisms

AT:ET: acceleration time: ejection time; LA: left atrium; LAP: left atrium pressure; LHD: left-heart disease; mPAP: mean pulmonary arterial pressure; PA: pulmonary artery; PAH: pulmonary arterial hypertension; PAWP: pulmonary arterial wedge pressure; PH: pulmonary hypertension; PVR: pulmonary vascular resistance; RA: right atrium; RHC: right heart catheterisation; RV: right ventricle; TRV: tricuspid regurgitation velocity; WU: Wood Units.

**Table 3:** Key mediators and signalling pathways in pulmonary hypertension.

Mediator/Pathway	Change in PH	Main Effect	Translational Value
**Endothelin-1**	↑	Vasoconstriction, smooth-muscle proliferation, fibrosis	Established therapeutic target in human PAH
**Nitric oxide-sGC-cGMP**	↓	Reduced vasodilation and antiproliferative signalling	Major therapeutic target in human PAH and in canine PH through PDE5 inhibition
**Prostacyclin**	↓	Reduced vasodilation, antiproliferative and antiplatelet effects	Established therapeutic target in human PAH
**BMP/TGF-β**	Dysregulated	Abnormal repair, proliferation, fibrosis, vascular remodelling	Key disease-modifying pathway. relevant to sotatercept
**Serotonin**	Dysregulated/↑	Vasoconstriction and smooth- muscle proliferation	Comparative mechanistic relevance in human and canine PH
**Growth factors (e.g., PDGF, FGF)**	↑	Cell proliferation, migration, matrix production	Emerging anti-remodelling target class
**Inflammatory mediators (e.g., IL-6, IL-1)**	↑	Endothelial injury, immune cell recruitment, fibroblast activation	Biomarker and therapeutic interest, but still investigational
**Platelet activation/thrombosis-related** **pathways**	↑	In situ thrombosis, endothelial injury, vascular obstruction	Particularly relevant in thromboembolic PH
**Metabolic dysfunction/mitochondrial** **remodelling**	Dysregulated	Apoptosis resistance, proliferation, maladaptive RV energetics	Emerging mechanistic and therapeutic target
**RV maladaptation**	↑	Hypertrophy, fibrosis, RV-PA uncoupling, RV failure	Major unmet therapeutic need across PH phenotypes

BMP: bone morphogenetic protein; cGMP: cyclic guanosine monophosphate; FGF: fibroblast growth factor; IL: interleukin; PAH: pulmonary arterial hypertension; PDGF: platelet-derived growth factor; PH: pulmonary hypertension; RV: right ventricle; RV-PA: right ventricle-pulmonary artery; sGC: soluble guanylate cyclase; TGF-β: transforming growth factor-β.

## Data Availability

No new data were created or analyzed in this study. Data sharing is not applicable to this article.

## Abbreviations

The following abbreviations are used in this manuscript:

5-HT	Serotonin
ACVIM	American College of Veterinary Internal Medicine
BMP	Bone morphogenetic protein
BNP	B-type natriuretic peptide
BOAS	Brachycephalic obstructive airway syndrome
CHF	Congestive heart failure
CIPF	Canine idiopathic pulmonary fibrosis
CT	Computed tomography
CTEPH	Chronic thromboembolic pulmonary hypertension
EndoMT	Endothelial-to-mesenchymal transition
HIV	Human immunodeficiency virus
HRCT	High-resolution computed tomography
ILD	Interstitial lung disease
IPF	Idiopathic pulmonary fibrosis (human)
LA	Left atrium/left atrial
LHD	Left-heart disease
MMVD	Myxomatous mitral valve disease
MMP	Matrix metalloproteinase
mPAP	Mean pulmonary arterial pressure
MR	Mitral regurgitation
MVP	Mitral valve prolapse
NT-proBNP	N-terminal pro-B-type natriuretic peptide
OSA	Obstructive sleep apnoea
PA	Pulmonary artery/pulmonary arterial
PAH	Pulmonary arterial hypertension
PAP	Pulmonary arterial pressure
PASMCs	Pulmonary arterial smooth-muscle cells
PAWP	Pulmonary arterial wedge pressure
PCH	Pulmonary capillary haemangiomatosis
PE/PT/PTE	Pulmonary embolism/pulmonary thrombosis/pulmonary thromboembolism
PH	Pulmonary hypertension
PVR	Pulmonary vascular resistance
PVOD	Pulmonary veno-occlusive disease
RA	Right atrium/right atrial
RHC	Right-heart catheterisation
RV	Right ventricle/right ventricular
RV-PA	Right ventricle–pulmonary artery (coupling)
SDB	Sleep-disordered breathing
SMC	Smooth-muscle cell
TGF-β	Transforming growth factor-β
TPH1	Tryptophan hydroxylase-1
TRV	Tricuspid regurgitation velocity
WBBP	Whole-body barometric plethysmography
WU	Wood units (mmHg·min·L^−1^)
